# Single-Cell Gene Expression Analysis of Cholinergic Neurons in the Arcuate Nucleus of the Hypothalamus

**DOI:** 10.1371/journal.pone.0162839

**Published:** 2016-09-09

**Authors:** Jae Hoon Jeong, Young Jae Woo, Streamson Chua, Young-Hwan Jo

**Affiliations:** 1 Division of Endocrinology, Department of Medicine, Albert Einstein College of Medicine, Bronx, United States of America; 2 Department of Genetics, Albert Einstein College of Medicine, Bronx, United States of America; 3 Department of Molecular Pharmacology, Albert Einstein College of Medicine, Bronx, United States of America; Nathan S Kline Institute, UNITED STATES

## Abstract

The cholinoceptive system in the hypothalamus, in particular in the arcuate nucleus (ARC), plays a role in regulating food intake. Neurons in the ARC contain multiple neuropeptides, amines, and neurotransmitters. To study molecular and neurochemical heterogeneity of ARC neurons, we combine single-cell qRT-PCR and single-cell whole transcriptome amplification methods to analyze expression patterns of our hand-picked 60 genes in individual neurons in the ARC. Immunohistochemical and single-cell qRT-PCR analyses show choline acetyltransferase (ChAT)-expressing neurons in the ARC. Gene expression patterns are remarkably distinct in each individual cholinergic neuron. Two-thirds of cholinergic neurons express tyrosine hydroxylase (*Th*) mRNA. A large subset of these Th-positive cholinergic neurons is GABAergic as they express the GABA synthesizing enzyme glutamate decarboxylase and vesicular GABA transporter transcripts. Some cholinergic neurons also express the vesicular glutamate transporter transcript gene. POMC and POMC-processing enzyme transcripts are found in a subpopulation of cholinergic neurons. Despite this heterogeneity, gene expression patterns in individual cholinergic cells appear to be highly regulated in a cell-specific manner. In fact, membrane receptor transcripts are clustered with their respective intracellular signaling and downstream targets. This novel population of cholinergic neurons may be part of the neural circuitries that detect homeostatic need for food and control the drive to eat.

## Introduction

The hypothalamus integrates circulating hormonal and nutritional signals to regulate energy homeostasis [1–4]. In particular, the arcuate nucleus of the hypothalamus (ARC) containing the melanocortin system plays a key role in the control of energy intake and expenditure [5–10]. In addition to the melanocortin system, there exists the cholinoceptive system in the ARC that is strongly linked to feeding behavior [11–13]. For instance, nicotine acts as a strong anorexigenic agent by activating β4-containing nicotinic receptors on proopiomelanocortin (POMC) neurons [13]. Both POMC and neuropeptide Y (NPY)/agouti-related peptide (AgRP) neurons are also downstream targets of cholinergic neurons [14]. Furthermore, the expression of *Agrp* and *Pomc* transcripts is regulated by the muscarinic acetylcholine (ACh) receptor type 3 [12].

As central cholinergic neurons are located mainly in the basal forebrain, laterodorsal & pedunculopontine tegmental nuclei, and recently dorsomedial hypothalamus [15, 16], it appears that cholinergic signal to ARC neurons originates in those structures. However, it has been described that a subpopulation of ARC neurons express cholinergic neuronal markers such as choline acetyltransferase (ChAT) and vesicular acetylcholine transporter (vAChT) in rats [17], consistent with the fact that there are local cholinergic neurons within the ARC. Importantly, POMC neurons express ChAT and vAChT, whereas cell bodies containing NPY/AgRP are distinct from vAChT-positive cells. These cholinergic neurons in the ARC may play a role in regulating feeding behavior.

In this study, we investigated 60 genes whose products are involved in peptide processing, fast neurotransmitter synthesis, neuronal activity, and intracellular signal transduction pathways in ARC neurons by combining single-cell real time quantitative PCR and single-cell whole transcriptome amplification methods. The rationale for investigating these genes is the following: (1) feeding-related neurons in the ARC express diverse membrane receptors that regulate neuronal activity [18–22]. These receptors are not randomly expressed but rather there is segregation of receptors such as serotonin, leptin, and insulin receptors in ARC POMC neurons [19, 23]. More importantly, their physiological functions appear to be distinct. For instance, leptin receptors on ARC neurons in particular POMC neurons regulate glucose homeostasis, but not food intake [24]. Insulin receptors on these neurons contribute to the control of locomotor activity and energy expenditure [25], whereas 5-HT_2C_ receptors on POMC neurons control both feeding and glucose homeostasis [26], (2) despite similar electrical outcomes of ARC neurons in response to circulating hormones, there are specific links between receptors and their respective enzyme isoforms. For instance, leptin and insulin depolarize POMC neurons via PLC γ isoform, whereas apelin-13 excites POMC neurons through activation of PLC β isoforms [18, 27, 28], (3) although all neurons contain GABA_A_ and glutamate receptors, each of ligand-gated channels has distinct roles in different neurons. For example, glutamatergic N-methyl-D-aspartate (NMDA) receptors in AgRP, but not POMC, neurons play a role in controlling energy balance [29] and different subtypes of NMDA receptors play distinct roles in synaptic plasticity [30], (4) diverse ionic channels, including M-type potassium (KCNQ), ATP-sensitive potassium (K_ATP_), and transient receptor potential-canonical (TRPC) channels contribute to the control of glucose and energy homeostasis [31–33]. Hence, single-cell analysis of our hand-picked gene expression will help understand the role of individual cells within the ARC neural network. We found that cholinergic neurons in the ARC were not neurochemically identical as they expressed enzymes responsible for the synthesis and release of GABA, glutamate, catecholamines, POMC-derived peptides as well as other neuropeptides.

## Materials and Methods

### Animals

All mouse care and experimental procedures were approved by the institutional animal care research advisory committee of the Albert Einstein College of Medicine. Mice used in these experiments included ChAT-IRES-Cre [34] and tdTomato reporter mice (The Jackson Laboratory). 2-month-old mice on a mixed C57BL6/129SVJ background were used for all experiments. Animals were housed in groups in cages under conditions of controlled temperature (22°C) with a 12:12 h light dark cycle and fed a standard chow diet with *ad libitum* access to water.

### Hypothalamic slice preparations

Transverse brain slices were prepared from 2 month-old ChAT-IRES-Cre::tdTomato mice as described in our prior study [35]. In brief, animals were anesthetized with isoflurane. After decapitation, brains were transferred into a sucrose-based solution bubbled with 95% O_2_/5% CO_2_ and maintained at ~3°C. This solution contained the following (in mM): 248 sucrose, 2 KCl, 1 MgCl_2_, 1.25 KH_2_PO_4_, 26 NaHCO_3_, 1 sodium pyruvate, and 10 glucose. Transverse coronal brain slices (200 μm) were prepared using a vibratome. Slices were maintained at 35°C and equilibrated with an oxygenated artificial cerebrospinal fluid (aCSF) containing the following (in mM): 113 NaCl, 3 KCl, 1 NaH_2_PO_4_, 26 NaHCO_3_, 2.5 CaCl_2_, 1 MgCl_2_, and 5 glucose in 95% O_2_/5% CO_2_.

### Immunocytochemistry

Mice were anesthetized with isoflurane and transcardially perfused with pre-perfusion solution (9 g NaCl, 5 g sodium nitrate, 1000 u heparin in 1L distilled water). Brains were immediately removed, postfixed in 4% PFA at 4°C overnight, and sectioned in 50 um with vibratome. Sections were permeabilized with 0.4% Triton X-100 and blocked with 1% BSA for 2 hrs at room temperature and then incubated with anti-ChAT (1:1000; Millipore, AB144P), anti-POMC (1:1000; Phoenix pharmaceuticals, H-029-30), and anti-Th (1:1000; Millipore, MAB318) antibodies diluted in blocking solution for 72 hrs at cold room. After incubation in primary antibodies, all sections were washed in PBS and then incubated with anti-goat IgG (1:500, A11055; Invitrogen), anti-rabbit IgG (1:500, A11008; Invitrogen), anti-mouse IgG(1:500, A11029; Invitrogen) diluted in blocking solution for 2 hrs at room temperature. Sections were then washed in PBS, dried and mounted with Vectashield mounting media. Images were acquired using a Leica confocal microscope.

### Single-cell real time qPCR and single-cell whole transcriptome amplification

Brain slices were placed on the stage of an upright, infrared-differential interference contrast microscope (Olympus BX50WI) mounted on a Gibraltar X-Y table (Burleigh) and visualized with a 40X water immersion objective using infrared microscopy. We collected cytoplasm containing total RNA from individual cholinergic neurons in slices via aspiration into glass pipette as described in our previous work [16, 18, 35]. The initial reverse transcription (RT) reaction was conducted after pressure ejection of the single cell samples into a microcentrifuge tube with REPLI-g WTA single cell kit (Qiagen). Samples were incubated for 10 min at 42°C with 2 μl gDNA wipeout buffer prior to addition of 7 μl RT mix to synthesize first strand cDNA (RT mix: 1 μl oligodT primer, 4 μl RT buffer, 1 μl random primer, 1 μl RT enzyme mix). The tubes were incubated at 42°C for 1 hr, and at 95°C for 3 min and then incubated at 24°C for another 30 min with 10 μl ligation mix (8 μl ligase buffer, 2 μl ligase Mix). The reaction was stopped by incubating at 95°C for 5 min. Samples were incubated for another 2 hrs at 30°C after adding the amplification mix (29 μl buffer, 1 μl REPLI-g SensiPhi DNA polymerase) and at 65°C for 5 min. Once reverse-transcribed cDNAs had been made, we purified cDNA using column based Fragment DNA Purification Kit (Cat # 17287, Intron Biotechnology Inc). And then, the quality and quantity of single cell cDNA were determined using a NanoDrop ND-1000 spectrophotometer (Thermo Scientific).

Single-cell qPCR was performed in sealed 96 well plates with SYBR Green master Mix in a Light Cycler. Single-cell qPCR reactions were prepared in a final volume of 20 μl containing 2 μl of single cell whole cDNA (10 ng) and 10 μl of SYBR Green master mix in the presence of primers at 0.5 μM. We first prepared standard curves for housekeeping genes (e.g. 18S ribosomal RNA (18S rRNA)) and target genes using a cDNA mixture made of a small amount (2 μl) of each sample. Cycle number was plotted against the normalized fluorescence intensity to visualize the PCR amplification and determine the amplification efficiencies.

To ensure the reliability and integrity of the single-cell qPCR and single-cell WTA assay, we used several types of controls; (1) we tested several reference genes such as β-actin, glyceraldehyde 3-phosphate dehydrogenase (GADPH), 18S rRNA, or green fluorescent protein (GFP) and found that the *18S rRNA* gene was most stable across cells, (2) all reactions were performed in triplicates, (3) the melting curves of each of the samples were checked to make sure that only one product was formed from the PCR reaction, (4) the PCR products were analyzed on agarose gels to further verify that the product size was correct and that there was only a single product, and (5) the amplification efficiencies of most primers tested were > 98% (S2 Table).

### Gene clustering and statistical analysis

Cycle Threshold (C_T_) values were extracted and melting curves of the C_T_ values were visually inspected for a quality control. The data was imported, normalized, and visualized using R 3.2.2 [36] and HTqPCR [37] package. ΔC_T_ normalization was carried out using C_T_ values of *18S rRNA* as a housekeeping gene. Normalized C_T_ values were visualized as heatmaps and hierarchical clustering results were added on top as dendrograms. Pearson correlation distances between samples or genes were used in the clustering analysis for column-dendrograms and row-dendrograms, respectively.

## Results

### The ARC contains cholinergic neurons

**Fig 1:**
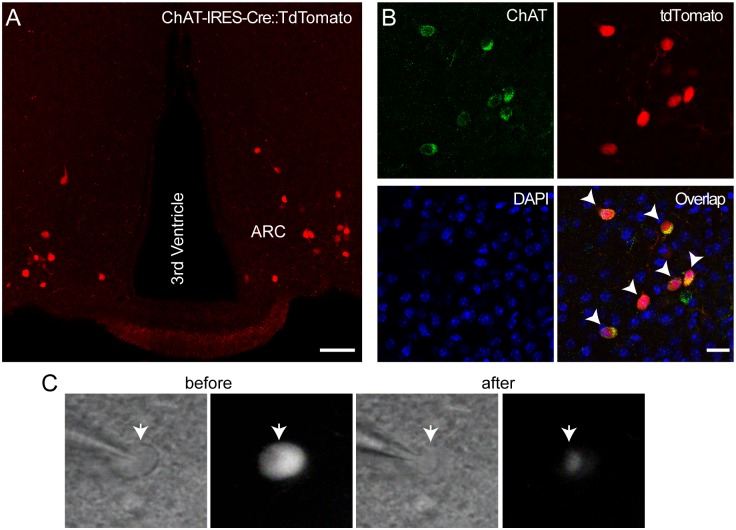
The ARC contains cholinergic neurons. **A and B.** Images of confocal fluorescence microscopy showing tdTomato-positive neurons in the ARC of ChAT-IRES-Cre::tdTomato mice (**A**). Immunostaining with anti-ChAT and anti-tdTomato antibodies showed co-labeling of ChAT and tdTomato (**B**, n = 7 animals). Arrow heads represent the neurons that were co-stained with these antibodies. Scale bars: 100 μm (left panel), 50 μm (right panel). **C.** Brightfield (left) and fluorescent (right) illumination of targeted cholinergic ARC neuron (white arrow) in hypothalamic slices of ChAT-IRES-Cre::tdTomato mice before and after collection of cytoplasm.

We crossed ChAT-IRES-Cre mice with tdTomato reporter mice to generate ChAT-IRES-Cre::tdTomato mice [34]. Fig 1A shows an example of tdTomato-positive neurons in the ARC from ChAT-IRES-Cre::tdTomato mice. Immunostaining with an anti-ChAT antibody revealed that approximately 90% of ChAT-positive neurons were positive to tdTomato (Fig 1B; n = 7 animals), consistent with the prior study showing that there exist cholinergic neurons in the ARC [17]. We investigated whether individual neurons in this specific population are molecularly and neurochemically identical by analyzing the expression of our hand-picked 60 genes involved in neuronal activity, peptide processing, neurotransmitter synthesis, and signal transduction pathways (S1 Table).

### ARC cholinergic neurons are neurochemically heterogeneous

**Fig 2:**
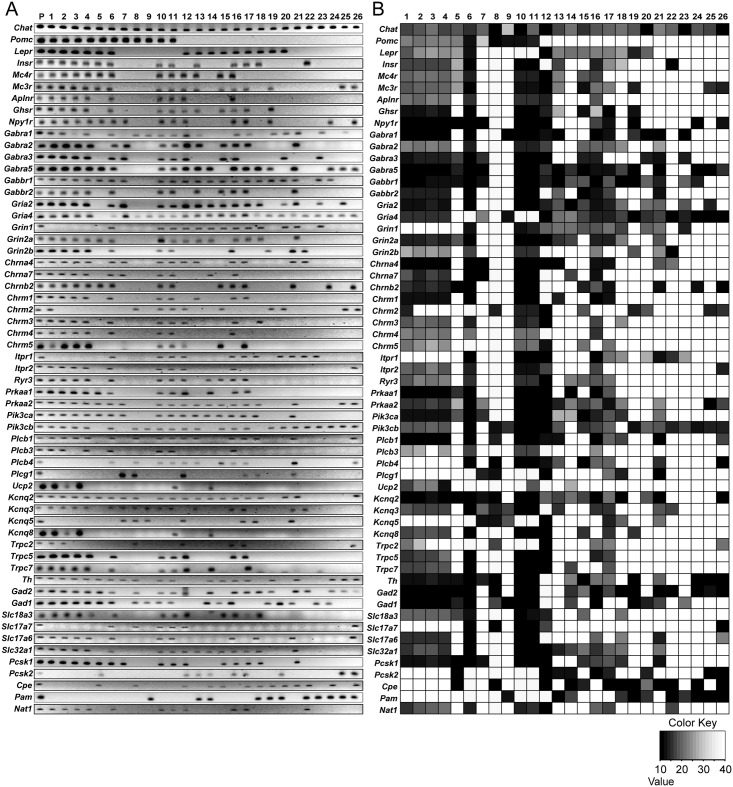
ARC cholinergic neurons are neurochemically heterogeneous. **A.** Gel image showing products of single-cell qRT-PCR following single-cell whole transcriptome amplification of cytoplasmic mRNA obtained from individual cholinergic neurons in mice. Transcripts were collected from 26 different cholinergic neurons from 10 different mice. **B.** Heatmap showing gene expression patterns across individual cholinergic neurons shown in Fig 2A. The transcript levels of each neuron were quantified using real-time qPCR.

We collected cytoplasm containing total RNA from individual cholinergic neurons in hypothalamic slices (Fig 1C). Single-cell qRT-PCR analysis revealed that cholinergic neurons in the ARC were neurochemically phenotypically distinct. Approximately two-thirds of cholinergic neurons in the ARC expressed tyrosine hydroxylase (*Th*) mRNA (n = 19 out of 26 neurons; Fig 2A and 2B). A large subpopulation of these TH-positive cholinergic neurons had the GABA synthesizing enzymes, including glutamic acid decarboxylase 65 (*Gad2*) and 67 (*Gad1*) (n = 18 and 13 out of 19 neurons, respectively) and vesicular GABA transporter (*Slc32a1*) transcript (n = 12 out of 19 neurons; Fig 2A and 2B). These findings are consistent with the recent study showing co-expression of dopamine and GABA in the ARC [38]. Interestingly, these cholinergic neurons expressed vesicular glutamate transporters in particular type 2 (*Slc17a6*, n = 11 and 19 neurons, respectively; Fig 2A and 2B).

**Fig 3:**
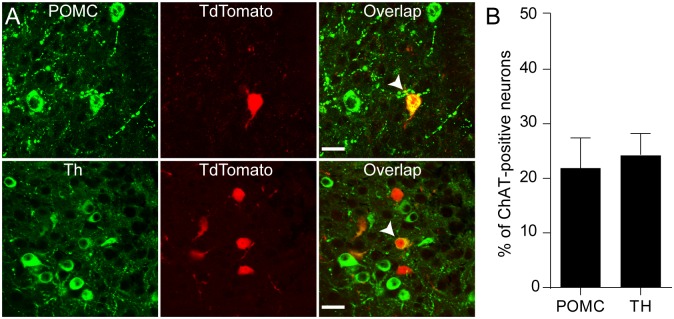
ARC cholinergic neurons express POMC and Th. **A.** Images of confocal fluorescence microscopy showing co-labelling of ChAT with POMC (upper panel) or Th (bottom panel). Scale bar: 50 μm. **B.** Summary plot showing that a subpopulation of cholinergic neurons expressed POMC or Th (21.91±5.5% or 24.34±3.9%; n = 3 animals, respectively).

We also found that 40% of ARC cholinergic neurons expressed the *Pomc* gene (n = 11 out of 26 neurons, Fig 2A and 2B), whereas no cholinergic neuron had *Npy* mRNA (n = 0 out of 14 neurons, data not shown). POMC processing enzymes such as prohormone convertase 2 (*Pcsk1*) and N-acetyltransferase-1 (*Nat1*) transcripts were detected in most cholinergic POMC neurons (n = 9 and 7 out of 11 neurons, respectively). These are consistent with the prior study showing co-expression of POMC and ChAT in the ARC [17]. However, other POMC processing enzymes, including prohormone convertase 2 (*Pcsk2*), carboxypeptidase E (*Cpe*) and peptidyl α-amidating monooxygenase (*Pam*) transcripts were expressed at extremely low levels below the detection threshold among these POMC-expressing cholinergic neurons (n = 1, 1, and 1 out of 11 neurons, respectively, Fig 2A and 2B). We also noted that cholinergic non-POMC neurons contained peptide processing enzymes such as *Pcsk1*, *Pcsk2*, *Cpe*, and *Pam* transcripts, suggesting that they have an ability to process neuropeptides that are not derived from POMC. We next performed immunocytochemistry with anti-POMC and anti-Th antibodies to examine if these cholinergic neurons indeed contain POMC and Th. We found that a subpopulation of cholinergic neurons was immune-positive to these antibodies (Fig 3A and 3B), further suggesting that some cholinergic neurons in the ARC contain POMC and Th.

### ARC cholinergic neurons are molecularly heterogeneous

Next we examined whether the expression of membrane receptors was heterogeneous as well. We found that leptin, insulin, and ghrelin receptor (*Lepr*, *Insr*, and *Ghsr*) transcripts were preferentially detected in Th-positive cholinergic neurons (n = 12, 10, and 12 out of 19 neurons, Fig 2A and 2B). Interestingly, TRPC2, 5 and 7 that are a downstream target of leptin and insulin receptors [27, 28] were located exclusively in cholinergic neurons having leptin or insulin receptors (*Trpc2*, *5*, *7* and *Lepr*, n = 11 out of 15 neurons; *Trpc2*, *5*, *7* and *Insr*, n = 9 out of 13 neurons, Fig 2A and 2B). M-type potassium channels (*Kcnq2*, *Kcnq3* and *Kcnq5*) regulated by apelin-13 [18] were detected in apelin receptor-expressing neurons (n = 9 out of 9 neurons, Fig 2A and 2B). In addition, cholinergic POMC neurons expressed nicotinic acetylcholine receptor subunits including α4 and α7 (*Chrna4* and *Chrna7*) and the muscarinic acetylcholine receptor type 3 mRNA (*Chrm3*). Cells expressing the muscarinic ACh receptor type 1(*Chrm1*) transcript also co-expressed with the downstream target KCNQ channels [39] (n = 10 out of 10 neurons, Fig 2A and 2B).

In contrast, transcripts for glutamate and GABA receptor subunits (*Gria2*, *Gria4*, *Grin1*, *Grin2a*, *Grin2b*, and *Gabra1*, *2*, *3 and 5*) did not cluster with other genes of interest in a biologically meaningful manner. Of note, more than half of cholinergic neurons co-expressed NPY receptor type 1 (*Npy1r*) and melanocortin receptor type 3 and 4 (*Mc3r* and *Mc4r*) transcripts (n = 15, 16, 12 out of 26 neurons, respectively).

### Gene clustering of leptin receptor-expressing cholinergic neurons in the ARC

**Fig 4:**
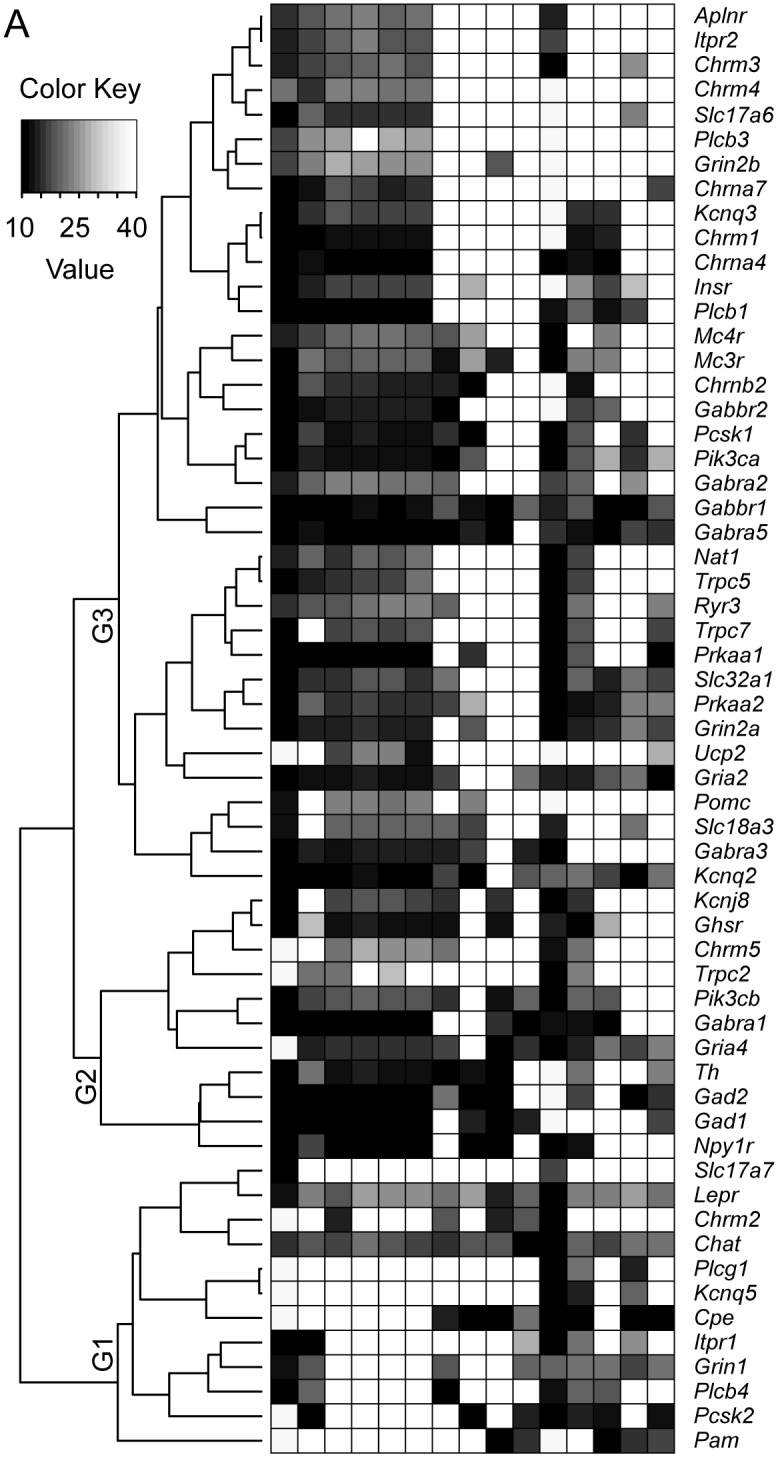
Gene clustering of leptin receptor-expressing cholinergic neurons in the ARC. **A.** Heatmap showing gene clustering in leptin receptor-positive cholinergic neurons. Genes were not randomly distributed across cells. For instance, the *Chat* gene clustered with peptide processing enzymes, including *Cpe*, *Pcsk2*, and *Pam* transcripts and the *Lepr* gene clustered with the PLCγ isoform (G1 gene group (G1)). The *Th* gene clustered with *Gad1*, *Gad2*, and *Npy1r* genes in the G2 gene group (G2). And *Pomc*, *Pcsk1*, and *Nat1* did not cluster together (G3 gene group (G3)).

We then examined whether genes with similar function would be more closely related than others using hierarchical clustering analysis. This shows that the *Lepr*-expressing neurons were subdivided into three groups of gene signatures and each cluster was represented by a genes of similar function (Fig 4). The first group of genes (G1 gene group) showed co-expression of peptide-processing enzymes, *Cpe*, *Pcsk2*, and *Pam* transcripts with the *Chat* gene (Fig 4). Within the second cluster (G2 gene group), The *Th* gene was co-expressed with GABA-synthesizing enzymes, *Gad2* and *Gad1*. On the other hand, *Pomc*, *Pcsk1*, and *Nat1* (an enzyme responsible for N-acetylation of α-MSH [40, 41]) were localized in the third cluster (G3 gene group) (Fig 4).

Hierarchical clustering further revealed that membrane receptor transcripts clustered with their respective intracellular signaling and downstream targets (i.e. *Aplnr*, *Plcβ*, and *Kcnq* transcripts [18]; *Lepr* and *Plcg* [27]; *Insr*, *Pik3ca*, and *Trpc* [28], *Chrm1* and *3*, *Plc*, and *Itpr* [42], Fig 4). Additionally, proteins that are functionally linked to one another were in the same group (Fig 4). For instance, 5' adenosine monophosphate-activated protein kinase α subunit (AMPK α) and uncoupling protein 2 (UCP2) that are required for glucose-sensing [32, 43] were in the same group (Fig 4).

## Discussion

In this study, we provide cellular evidence for molecular and neurochemical heterogeneity of cholinergic neurons in the ARC with an approach by combining single-cell real time quantitative PCR and single-cell whole transcriptome amplification methods. We found that the ARC contained cholinergic neurons. These cholinergic neurons were neurochemically distinct as they had the ability to synthesize and release diverse peptides, amines, and neurotransmitters. Approximately 70% of these cholinergic neurons expressed the *Th* gene. Th-positive cholinergic neurons contained Gad65/67 and vesicular GABA transporter transcripts, consistent with the recent study showing co-expression of dopamine and GABA in neurons in the ARC [38]. In addition, a subset of GABA-expressing cholinergic neurons had vesicular ACh as well as glutamate transporter transcripts. Gene clustering analysis further showed that membrane receptor transcripts clustered with their respective intracellular signaling and downstream targets.

The cholinoceptive system in the ARC plays a role in regulating food intake [11–13]. Activation of nicotinic acetylcholine receptors reduces energy intake via modulation of melanocortinergic neurons in the ARC [13, 14]. Both POMC and NPY neurons in the ARC are innervated by cholinergic input [14], implying that endogenous ACh can influence the activity of these neurons. In fact, mice lacking the M3 muscarinic ACh receptor consume less food and have lower body weights [12, 44]. The M3 receptor is expressed in the ARC and oppositely regulates *Pomc* and *Agrp* mRNA expression [44]. Hence, it appears that stimulation of hypothalamic nicotinic and muscarinic receptor-mediated components results in opposing physiological responses in part through the melanocortinergic system. It has been reported that a small subset of POMC neurons expressed ChAT and vAChT in rats [17]. Our single-cell qRT-PCR and immunohistochemical studies of ARC neurons also revealed the co-expression of POMC and ChAT in mice, suggesting that there are local cholinergic neurons in the ARC. Therefore, this novel population of neurons may be part of the neural circuitries that detect homeostatic need for food and control the drive to eat.

Most hypothalamic neurons contain multiple neuropeptides and/or neurotransmitters [45]. Within the ARC, Npy/AgRP neurons have GABA as a fast neurotransmitter and GABA released from Npy/AgRP neurons is involved in ghrelin-mediated feeding [46]. It has been also shown that AgRP, Npy, and GABA in these neurons play temporally distinct roles in driving food intake [47]. Of particular interest is that α-MSH and β-endorphin derived from the same precursor POMC have opposite effects on feeding. For instance, β-endorphin released from the same population of POMC neurons promotes feeding [48], while α-MSH release reduces food intake [49]. These findings suggest that, although the expression of distinct peptides and neurotransmitters in the same population appears to be a paradox, these transmitters and peptides could independently but cooperatively influence feeding. Likewise, multiple neurotransmitters and peptides expressed in cholinergic neurons can contribute to the control of feeding behavior.

Although single-cell gene expression profiling showed heterogeneity in gene expression within the same population, gene expression patterns in individual cholinergic cells appear to be highly regulated in a cell-specific manner. For instance, TRPC2, TRPC5 and TRPC7 channels that are a downstream target of leptin and insulin receptors [27, 28] were detected in cholinergic neurons with leptin and insulin receptors. Apelin-sensitive KCNQ potassium channels [18] were found in apelin receptor-expressing neurons. The muscarinic ACh receptor type 1 was also co-detected with its downstream target KCNQ channels [39]. In line with these findings, the membrane receptors also clustered with their respective signaling pathways (i.e. apelin receptor—PLCβ, [18]; leptin receptor—PLCγ [27]; Insulin receptor—PI3K 110α [28], mAChR1 and 3—PLC—IP3R [42]). In addition, genes that are functionally linked to one another were clustered in the same group (i.e. AMPK α and UCP2 [32, 43]. All UCP2-expressing cholinergic POMC neurons co-expressed K_ATP_ channels, which require for glucose-sensing in POMC neurons [32].

The experimental methodology described in this study has some technical advantages over Fluorescence Activated Cell Sorting (FACS) technique. First, our single-cell whole transcriptome amplification of total cytoplasmic RNA from individual neurons can yield approximately 20 μg cDNA /μl. This is larger than traditional single cell RT-PCR protocol practiced previously [50] and is a sufficient amount to quantify transcript levels of thousands of genes for each cell. It should be noted that the amounts produced are sufficient for whole transcriptome profiling by methods like RNASeq. Second, this method has the ability to determine gene expression profiles of retrogradely and/or anterogradely labeled neurons as the small number of fluorescently labeled neurons is sufficient. Third, this approach may be useful for discovering therapeutic targets. In fact, many drugs are designed to disrupt receptor functions, specific enzyme isoforms, or their respective downstream targets in order to impact disease relevant molecular pathways only. These targeted therapies may require identification of specific disease biomarkers at a single cell level.

Our present study demonstrates a novel population of cholinergic neurons in the ARC. Individual cholinergic neurons were neurochemically heterogeneous as they expressed enzymes responsible for the synthesis and release of GABA, glutamate, catecholamines, POMC-derived peptides as well as other neuropeptides. Despite this heterogeneity, gene expression patterns in individual cholinergic cells appear to be highly regulated in a cell-specific manner. In fact, membrane receptor transcripts clustered with their respective intracellular signaling and downstream targets. Therefore, our present study has a potential in establishing links between the molecular identities at a neuronal level and their physiological functions within a neural network.

## Supporting Information

S1 Table Genes that are analyzed in the study. (DOCX)

S2 Table Primer sets for single cell qRT-PCR. (DOCX)
