# Supplementary material for: Single-Cell Gene Expression Analysis of Cholinergic Neurons in the Arcuate Nucleus of the Hypothalamus
Source: PLoS One. 2016 Sep 9;11(9):e0162839. doi: 10.1371/journal.pone.0162839 (PMC5017726; doi:10.1371/journal.pone.0162839)
Supplement: S1 Table — (DOCX) [file pone.0162839.s001.docx]

| **Gene Description** | **Gene Symbol** | **NCBI Reference Sequence** |
| --- | --- | --- |
| choline acetyltransferase | *Chat* | NM_009891.2 |
| pro-opiomelanocortin | *Pomc* | NM_001278581.1 |
| Leptin Receptor | *Lepr* | NM_001122899.1 |
| Insulin Receptor | *Insr* | NM_010568.2 |
| MC4 Receptor | *Mc4r* | NM_016977.4 |
| MC3 Receptor | *Mc3r* | NM_008561.3 |
| Apellin Receptor | *Aplnr* | NM_011784.3 |
| growth hormone secretagogue receptor | *Ghsr* | NM_177330.4 |
| NPY receptor type 1 | *Npy1r* | NM_010934.4 |
| GABAa R alpha1 | *Gabra1* | NM_010250.5 |
| GABAa R alpha2 | *Gabra2* | NM_008066.3 |
| GABAa R alpha3 | *Gabra3* | NM_008067.4 |
| GABAa R alpha5 | *Gabra5* | NM_176942.4 |
| GABAb R type 1 | *Gabbr1* | NM_019439.3 |
| GABAb R type 2 | *Gabbr2* | NM_001081141.1 |
| glutamate receptor, ionotropic, AMPA2 (alpha 2) | *Gria2* | NM_001039195.1 |
| glutamate receptor, ionotropic, AMPA4 (alpha 4) | *Gria4* | NM_001113180.1 |
| glutamate receptor, ionotropic, NMDA1 (zeta 1) | *Grin1* | NM_008169.3 |
| glutamate receptor, ionotropic, NMDA2A (epsilon 1) | *Grin2a* | NM_008170.2 |
| glutamate receptor, ionotropic, NMDA2B (epsilon 2) | *Grin2b* | NM_008171.3 |
| cholinergic receptor, nicotinic, alpha polypeptide 4 | *Chrna4* | NM_015730.5 |
| cholinergic receptor, nicotinic, alpha polypeptide 7 | *Chrna7* | NM_007390.3 |
| cholinergic receptor, nicotinic, beta polypeptide 2 | *Chrnb2* | NM_009602.4 |
| cholinergic receptor, muscarinic 1 | *Chrm1* | NM_001112697.1 |
| cholinergic receptor, muscarinic 2 | *Chrm2* | NM_203491.3 |
| cholinergic receptor, muscarinic 3 | *Chrm3* | NM_033269.4 |
| cholinergic receptor, muscarinic 4 | *Chrm4* | NM_007699.2 |
| cholinergic receptor, muscarinic 5 | *Chrm5* | NM_205783.2 |
| inositol 1,4,5-trisphosphate receptor 1 | *Itpr1* | NM_010585.5 |
| inositol 1,4,5-triphosphate receptor 2 | *Itpr2* | NM_010586.2 |
| ryanodine receptor 3 | *Ryr3* | NM_177652.2 |
| protein kinase, AMP-activated, alpha 1 catalytic subunit | *Prkaa1* | NM_001013367.3 |
| protein kinase, AMP-activated, alpha 2 catalytic subunit | *Prkaa2* | NM_178143.2 |
| phosphatidylinositol 3-kinase, catalytic, alpha polypeptide | *Pik3ca* | NM_008839.2 |
| phosphatidylinositol 3-kinase, catalytic, beta polypeptide | *Pik3cb* | NM_029094.3 |
| phospholipase C, beta 1 | *Plcb1* | NM_001145830.1 |
| phospholipase C, beta 3 | *Plcb3* | NM_001290349.1 |
| phospholipase C, beta 4 | *Plcb4* | NM_013829.2 |
| phospholipase C, gamma 1 | *Plag1* | NM_021280.3 |
| Uncoupling Protein 2 | *Ucp2* | NM_011671.4 |
| potassium voltage-gated channel, subfamily Q, member 2 | *Kcnq2* | NM_001003824.2 |
| potassium voltage-gated channel, subfamily Q, member 3 | *Kcnq3* | NM_152923.2 |
| potassium voltage-gated channel, subfamily Q, member 5 | *Kcnq5* | NM_001160139.1 |
| potassium inwardly-rectifying channel, subfamily J, member 8 | *Kcnj8* | NM_008428.4 |
| Tyrosine hydroxylase | *Th* | NM_009377.1 |
| transient receptor potential channel, subfamily C, member 2 | *Trpc2* | NM_001109897.2 |
| transient receptor potential channel, subfamily C, member 5 | *Trpc5* | NM_009428.2 |
| transient receptor potential channel, subfamily C, member 7 | *Trpc7* | NM_012035.2 |
| glutamic acid decarboxylase 2 | *Gad2* | NM_008078.2 |
| glutamic acid decarboxylase 1 | *Gad1* | NM_008077.4 |
| solute carrier family 18 (vesicular monoamine), member 3 | *Slc18a3* | NM_021712.2 |
| solute carrier family 17, member 7 | *Slc17a7* | NM_182993.2 |
| solute carrier family 17, member 6 | *Slc17a6* | NM_080853.3 |
| solute carrier family 32 (GABA vesicular transporter), member 1 | *Slc32a1* | NM_009508.2 |
| proprotein convertase subtilisin/kexin type 1 | *Pcsk1* | NM_013628.2 |
| proprotein convertase subtilisin/kexin type 2 | *Pcsk2* | NM_008792.4 |
| carboxypeptidase E (CPE) | *Cpe* | NM_013494.3 |
| peptidylglycine alpha-amidating monooxygenase | *Pam* | NM_013626.3 |
| N-acetyl transferase 1 | *Nat1* | NM_008673.1 |
