# Supplementary material for: Single-Cell Gene Expression Analysis of Cholinergic Neurons in the Arcuate Nucleus of the Hypothalamus
Source: PLoS One. 2016 Sep 9;11(9):e0162839. doi: 10.1371/journal.pone.0162839 (PMC5017726; doi:10.1371/journal.pone.0162839)
Supplement: S2 Table — (DOCX) [file pone.0162839.s002.docx]

| Primer | Forward | Reverse | Product | Annealing Temp. | Slope | Efficiency |
| --- | --- | --- | --- | --- | --- | --- |
| *Chat* | CACCAACAGCAAAGGAAAGA | GGATGGGAGCAGGGTTAGTA | 110 bp | 60 | -3.331 | 0.995 |
| *Pomc* | CCATAGATGTGTGGAGCTGGTG | CATCTCCGTTGCCAGGAAACAC | 138 bp | 60 | -3.367 | 0.981 |
| *Lepr* | AAGTTGTGGTTTGGGCTGAT | GGCAATCTGACCTGAGTGTG | 116 bp | 62 | -3.401 | 0.967 |
| *Insr* | GAGACCTTGGAAATTGGGAA | GATGGTGAGGTTGTGTTTGC | 101 bp | 60 | -3.32 | 0.999 |
| *Mc4r* | ATCATGTGTAACGCCGTCAT | GATGCCTCCCAGAGGATAGA | 105 bp | 60 | -3.339 | 0.990 |
| *Mc3r* | CACCTGGTCCTCATCATCAC | ATGACGGAGTTGCACATGAT | 101 bp | 60 | -3.374 | 0.977 |
| *Aplnr* | ACTTCTTCATTGCCCAAACC | GGCAAAGGTCACTACAAGCA | 113 bp | 60 | -3.309 | 1.004 |
| *Ghsr* | GGAAAACAGATATCTTCCCACG | GGGACCAGAACCACAAACAG | 101 bp | 60 | -3.392 | 0.972 |
| *Npy1r* | GTCTCTGGAAACCTGGCATT | GTGAACGGGAGACACATGAC | 128 bp | 60 | -3.385 | 0.972 |
| *Gabra1* | TGCTGGACGGTTATGACAAT | GAAACTGGTCCGAAACTGGT | 97 bp | 60 | -3.361 | 0.981 |
| *Gabra2* | GCTCTCTGCTACAGCTTCCA | ACTGCAGCAGCCTAAAGACC | 66 bp | 60 | -3.333 | 0.995 |
| *Gabra3* | AAATTTCCCGCATCATCTTC | GTTGCTGTGCTGCCACTATT | 132 bp | 60 | -3.351 | 0.986 |
| *Gabra5* | TCCATTGCACACAACATGAC | GCAGAGATTGTCAGACGCAT | 92 bp | 60 | -3.39 | 0.972 |
| *Gabbr1* | TCCTTCCATCATCCTTCTCC | TGTGTGTGTGTGGTGAGTCC | 86 bp | 60 | -3.411 | 0.963 |
| *Gabbr2* | CCTCTCCAGACTGCTGGAAT | AGTCATTGCCATTCCTCTCC | 108 bp | 60 | -3.331 | 0.995 |
| *Gria2* | ACGCTCTACCACCTTACCCA | TTTGCTTCTGTGGCAACTCT | 118 bp | 60 | -3.327 | 0.995 |
| *Gria4* | GAAATGCTGTTAACCTCGCA | CTGCCACATTCTCCTTTGTC | 97 bp | 60 | -3.344 | 0.990 |
| *Grin1* | TTCTCTCCTGCTGCACAATC | CACTAGGGCAGTGAAGGCTA | 95 bp | 60 | -3.357 | 0.986 |
| *Grin2a* | GGGATGACCAACGCTTAGTT | CCTCAAGGATGACCGAAGAT | 104 bp | 60 | -3.362 | 0.982 |
| *Grin2b* | GGCTTGGCTCTATGGAGAAG | TCTTTGCAGAGCAGAAGGAA | 103 bp | 60 | -3.381 | 0.977 |
| *Chrna4* | GAGGCCTGGTCTCTGAACTC | GACTGAAACATGGCAGATGG | 88 bp | 60 | -3.328 | 0.995 |
| *Chrna7* | AGATGGCCAGATTTGGAAAC | CCAGATGCATTCACCAAGAC | 99 bp | 60 | -3.318 | 0.999 |
| *Chrnb2* | CTTGGGAAAGGTGAGGTGTT | CTGTAACATGGCTCCCAATG | 119 bp | 60 | -3.361 | 0.986 |
| *Chrm1* | CCAAAAGCTCCCCAAATACA | TCTCTTGGCCAGTTGTTCCT | 101 bp | 60 | -3.101 | 1.098 |
| *Chrm2* | GGAGCACAACAAGATCCAGA | CTGACAGACGTGGAGTCGTT | 105 bp | 60 | -3.358 | 0.986 |
| *Chrm3* | ACAGCTGCATACCCAAAACC | TCTTGTTGCACAGGGCATAG | 96 bp | 60 | -3.314 | 0.999 |
| *Chrm4* | AGATGGTGTTCATTGCGACA | CAACTGCCTGTTGACCTTGA | 98 bp | 60 | -3.279 | 1.018 |
| *Chrm5* | ACCTCAGCCATCAAATGACC | GATGATGAAGGCCAGGAGAA | 98 bp | 60 | -3.292 | 1.013 |
| *Itpr1* | CTCTGACCTCTGCGTATCCA | CAGCTTGGTCTCAATCAGGA | 109 bp | 60 | -3.381 | 0.977 |
| *Itpr2* | ATCGATAGGCCTTGGATTTG | TCCTGTCTCGATGACCTGAG | 99 bp | 60 | -3.338 | 0.990 |
| *Ryr3* | ACCTCTGTGACTGCGAACTG | TGAGCTCATTGTAGCGGAAC | 108 bp | 60 | -3.377 | 0.977 |
| *Prkaa1* | TCGCCTACCACCTCATCATA | GTGAGGCCGAGTTAAATGGT | 116 bp | 65 | -3.315 | 0.995 |
| *Prkaa2* | CTCCCAGGGTGAAGTCTCAT | CTGAAGGACAAGTTGCCAGA | 90 bp | 65 | -3.341 | 0.991 |
| *Pik3ca* | ACTGCGTGGCAACCTTTATC | TCCAAAAAGTGCCCAAAATC | 106 bp | 65 | -3.359 | 0.986 |
| *Pik3cb* | ATCAGACCATCAGGAAAGCC | GCATTTCTTCCAGCTCATCA | 144 bp | 65 | -3.392 | 0.972 |
| *Plcb1* | CCTGTTTGAGGATAGCAGCA | TAGCCTGCACAGGCAATATC | 90 bp | 62 | -3.394 | 0.972 |
| *Plcb3* | GTGTGGAGCTGGATGTATGG | ACCTCAGTGGTCATGGTGAA | 82 bp | 65 | -3.382 | 0.977 |
| *Plcb4* | TCCTTCCTTTGGATGGTCTC | TCCATCAGGCACGTATGTTT | 125 bp | 65 | -3.336 | 0.995 |
| *Plag1* | AATCAAGGAGATCCGTCCAG | AGCGGAATTCCATTCCATAG | 119 bp | 65 | -3.361 | 0.977 |
| *Ucp2* | CCTACAAGACCATTGCACGA | CATAGGTCACCAGCTCAGCA | 108 bp | 60 | -3.392 | 0.972 |
| *Kcnq2* | TGTGTTATGCGGTTCTTGGT | TGTCCAGCCGAGTACTGTTC | 95 bp | 65 | -3.349 | 0.990 |
| *Kcnq3* | GAGGAACAACGCCAAGTACA | AGAATCAAGCATCCCAGGAC | 123 bp | 65 | -3.352 | 0.986 |
| *Kcnq5* | ATCTCAAGAGGCCTGCAGTT | ATGGGTACCTGGGTAGCTTG | 104 bp | 65 | -3.371 | 0.982 |
| *Kcnj8* | ACGTGATTGACAAGCGTAGC | ACGCCCTCGAGAATCACTAT | 91 bp | 60 | -3.396 | 0.968 |
| *Th* | AGTGCCAGAGAGGACAAGGT | GTCCAGGTCAGGGTCAAACT | 96 bp | 60 | -3.328 | 0.995 |
| *Trpc2* | TGCTGTGGAAGTCAGATGGT | CCAGAACTCAAAGCAAGCTG | 120 bp | 60 | -3.413 | 0.963 |
| *Trpc5* | CCAAAACAAATGAGGGGCTA | CCTGTTTCCCAAGAGGTCAA | 98 bp | 60 | -3.316 | 1.004 |
| *Trpc7* | CACAGAAGAGGTGGAAGCAA | TTTGATTCGGCTCAGACTTG | 88 bp | 60 | -3.374 | 0.977 |
| *Gad2* | TAGTTTGCGCACTCTGGAAG | TGACCATTGTGGTCCCATAC | 104 bp | 60 | -3.361 | 0.986 |
| *Gad1* | ACATCGACTGCCAATACCAA | CCATCCAACGATCTCTCTCA | 102 bp | 60 | -3.366 | 0.982 |
| *Slc18a3* | GGTGGCTAAGCCTTTCTCAG | CTAGCATGAGGCGATGGATA | 94 bp | 60 | -3.367 | 0.982 |
| *Slc17a7* | GTCTATGCCATCATCGTTGC | CAAAGCCGAACACTTCTTCA | 97 bp | 60 | -3.386 | 0.972 |
| *Slc17a6* | ATTGGAGAGAGCGCAAATCT | CTGCAGAAGTTGGCAACAAT | 110 bp | 60 | -3.335 | 0.995 |
| *Slc32a1* | ATGTGGCCATCTTCGTCAT | CCTAGTCCTCTGCGTTGGTT | 104 bp | 60 | -3.334 | 0.995 |
| *Pcsk1* | GTGGAGGGTAGAAGGGATGA | TGAGCTTTGCACTTGGAGAC | 130 bp | 60 | -3.337 | 0.995 |
| *Pcsk2* | CAACTGTGACGGCTATGCTT | TGCTGAAGGTGGATGCTAAG | 119 bp | 60 | -3.336 | 0.995 |
| *Cpe* | TGCATGGTAATGAGGCGGTT | GGTGCTGTGGATCAGGTTGA | 110 bp | 60 | -3.393 | 0.968 |
| *Pam* | GGAGCTGTCTTCGTGTCAGA | GGACTGCTCCCAGAAGACTC | 107 bp | 62 | -3.357 | 0.986 |
| *Nat1* | CAGATGCGAGCAGTTCCTT | CCATCCACCTCTCTTCTTCC | 111 bp | 60 | -3.391 | 0.972 |
| 18S rRNA | CTCAACACGGGAAACCTCAC | CGCTCCACCAACTAAGAACG | 98 bp | 55 | -3.381 | 0.972 |
